# Effects of an astaxanthin‐containing supplement on oxidative status in skeletal muscle and circulation during deconditioning and reconditioning periods in polo ponies

**DOI:** 10.14814/phy2.70346

**Published:** 2025-04-26

**Authors:** Mia Y. Kawaida, Oh. Sung Kwon, Ahram Ahn, Amanda S. Reiter, Nicole M. Tillquist, Sung Gi Noh, Jung W. Lee, Timothy E. Moore, Sarah A. Reed

**Affiliations:** ^1^ Department of Animal Science University of Connecticut Storrs Connecticut USA; ^2^ Department of Kinesiology University of Connecticut Storrs Connecticut USA; ^3^ Department of Orthopaedic Surgery & Center on Aging University of Connecticut School of Medicine Farmington Connecticut USA; ^4^ Department of Statistics University of Connecticut Storrs Connecticut USA; ^5^ Statistical Consulting Services, Center for Open Research Resources & Equipment University of Connecticut Storrs Connecticut USA; ^6^ Present address: Department of Applied Physiology and Kinesiology University of Florida Gainesville USA; ^7^ Present address: Department of Animal Science Tarleton State University Stephenville USA

**Keywords:** astaxanthin, deconditioning, mitochondria, oxidative status, reconditioning

## Abstract

This study investigated the effects of astaxanthin (ASTX) supplementation on oxidative status during a deconditioning‐reconditioning cycle. Twelve polo ponies were assigned to no supplementation (CON) or an ASTX supplemented group, which received oral administration of a supplement containing 75 mg ASTX daily for 32 weeks. Polo ponies underwent a 16‐week deconditioning period (DECON) followed by a 16‐week reconditioning program (RECON). Submaximal exercise tests (SETs) were performed at the beginning of the study (Baseline), after DECON, and after RECON. Blood samples were collected at −30, 0, 15, 30, and 60 min relative to each SET for oxidative status analysis. Muscle samples were collected 2 weeks before (Pre‐Ex) and 2 h after (Post‐Ex) each SET for muscle oxidative status and gene expression analyses. Pre‐Ex muscles were analyzed for high‐resolution respirometry. Circulating glutathione peroxidase (GPX) activity was increased (*p* ≤ 0.02) and protein carbonylation was decreased in ASTX (*p* ≤ 0.05). Muscle oxidative status was affected by DECON and reconditioning (*p* ≤ 0.05). ASTX increased gene expression of PPARGC1A after reconditioning (*p* ≤ 0.05). Deconditioning reduced oxidative phosphorylation at complex I and II (*p* = 0.01). Thus, a deconditioning‐reconditioning cycle had greater impacts on muscle oxidative capacity than ASTX supplementation.

## 1 INTRODUCTION

Regular exercise training or conditioning can induce metabolic and physiological adaptations in circulation, skeletal muscle, and mitochondria, leading to enhanced athletic performance. One of these adaptations is an improved ability to mitigate oxidative stress that occurs during exercise. Conditioning may increase circulating and muscle antioxidant capacity through greater antioxidant activities, such as increased superoxide dismutase (SOD) and glutathione peroxidase (GPX) activities, which scavenge free radicals produced in the skeletal muscle during exercise (Fatouros et al., 2004; Powers et al., 1994). Accordingly, conditioning may attenuate oxidative damage to lipids, proteins, and DNA in circulation and muscle, indicated by a reduction in malondialdehyde (MDA), protein carbonylation, and 8‐hydroxy‐2′‐deoxyguanosine (8‐OHdG) concentrations, respectively (Gram et al., 2015; Pingitore et al., 2015; Radák et al., 2002; Silva et al., 2009). Additionally, skeletal muscle can gain greater oxidative capacity following conditioning through mitochondrial adaptations. Muscle oxidative capacity heavily relies on mitochondrial content. Conditioning could promote mitochondrial biogenesis by inducing transcriptional factors and cofactors like peroxisome proliferator‐activated receptor gamma coactivator‐1 alpha (PGC‐1α; (Buso et al., 2019; Ferraro et al., 2014; Vargas‐Mendoza et al., 2021)). As a result, mitochondrial content and size increase, leading to greater mitochondrial respiratory capacity and therefore greater muscle oxidative capacity (Ferraro et al., 2014; Holloszy, 1967). Thus, conditioning increases both the ability of mitochondria to produce ATP and the ability to mitigate increased oxidative stress.

Periods of deconditioning, however, can cause a partial or complete reversal of these conditioning‐induced adaptations. Deconditioning may decrease circulating and muscle SOD and GPX activities (Lawler et al., 2003), leading to the accumulation of oxidized proteins and lipids (Lawler et al., 2003; Powers et al., 2012) and therefore greater oxidative stress (Bradic et al., 2018; Park & Kwak, 2016; Rai et al., 2010). Moreover, muscle oxidative capacity may decrease due to alterations in muscle morphometry, enzyme activity, and gene expression. Mitochondrial volume and density decline (Ferretti et al., 1997; Nielsen et al., 2010) via downregulation of PGC‐1α (Abadi et al., 2009; Buso et al., 2019). Further, the activity of enzymes associated with oxidative metabolism, for example, succinate dehydrogenase, can decrease after deconditioning (Abadi et al., 2009; Jansson et al., 1988). These alterations reduce mitochondrial respiration and consequently muscle oxidative capacity. Taken together, periods of deconditioning may not only decrease physiological capacity but also abolish adaptations resulting from conditioning, which may compromise athletic performance.

Protection from excessive reactive oxygen species (ROS) production during exercise is regulated by the antioxidant defense system through reduction pathways (Kirschvink et al., 2008), which may be further improved by exogenous antioxidants, such as supplemental dietary antioxidants (Parker et al., 2014). For instance, astaxanthin (ASTX), which belongs to a family of keto‐carotenoids and is produced only in yeast and microalgae (Harith et al., 2020), has the greatest antioxidant activity among all the natural sources of carotenoids (Ambati et al., 2014; Goto et al., 2001). With polar terminal rings containing hydroxyl and carboxyl groups and a nonpolar long lipophilic chain, ASTX locates intermolecularly and intramolecularly in the phospholipid membrane, allowing free radicals to be trapped at the surface and inside of the membrane (Goto et al., 2001). Use of ASTX as a nutritional supplement mitigates oxidative stress by inhibiting ROS production in skeletal muscle of men, rats, and mice (Baralic et al., 2013; Chen et al., 2021; Yu et al., 2022). In horses, supplementation of ASTX together with L‐carnitine decreases muscle damage (Sato et al., 2015). In human cancer cells, ASTX increases oxygen consumption (Wolf et al., 2010). Astaxanthin also stimulates mitochondrial biogenesis by upregulating PGC‐1α (Cardanho‐Ramos & Morais, 2021; Nishida et al., 2021). Acting as an indirect antioxidant, ASTX enhances plasma SOD and GPX activities as well as total antioxidant capacity (Choi et al., 2011; McAllister et al., 2022). Correspondingly, oxidative damage to lipids, proteins, and DNA is reduced in individuals supplemented with ASTX (Park et al., 2010; Petyaev et al., 2018; Sztretye et al., 2019; Wolf et al., 2010). These properties of ASTX lead to improved exercise performance in supplemented individuals by increasing endurance capacity (Aoi et al., 2008; Ikeuchi et al., 2007; Nakanishi et al., 2022; Polotow et al., 2014).

Often, periods of deconditioning are followed by reconditioning periods to gradually reintroduce the body to physical activity. Importantly, how this deconditioning and reconditioning cycle affects the oxidative status of skeletal muscle and circulation, or if exogenous antioxidant supplementation can improve the response to deconditioning and reconditioning, is poorly understood. Currently, there is limited understanding of whether ASTX supplementation during deconditioning may mitigate the loss of antioxidant protection or improve protective effects during subsequent retraining. Thus, the objective of this study was to determine if ASTX supplementation improves oxidative status during deconditioning and reconditioning periods in horses. We hypothesized that (1) supplementation with ASTX would increase antioxidant capacity and decrease oxidative stress in the muscle and circulation in response to exercise following deconditioning and reconditioning periods, (2) horses supplemented with ASTX would have greater resting mitochondrial respiration, and (3) ASTX supplementation would upregulate the expression of genes involved in antioxidant activities and mitochondrial respiration and biogenesis.

## 2 MATERIALS AND METHODS

### 2.1 Animals

This study was reviewed and approved by the Institutional Animal Care and Use Committee (IACUC) at the University of Connecticut (A19‐056). Twelve polo ponies, horses used to play polo, including 10 mares and two geldings (*n* = 12) with a mean age of 14.0 ± 1.6 years (range: 7–22 years) and body weight (BW) of 488.3 ± 10.6 kg (range: 444.5–581.4 kg) were used in the study. The horses were weighed at the beginning of the study, following a deconditioning period (DECON), and following a reconditioning period (RECON). There were no observed effects of treatment, conditioning status, or interaction on BW (*p* ≥ 0.72). One horse was removed from the study after DECON due to a suspensory ligament injury unrelated to the study. Data collected from this horse at Baseline and DECON were retained in the data set. Horses were individually housed in the University of Connecticut Lorentzon Stables.

### 2.2 Deconditioning and reconditioning

**FIGURE 1:**
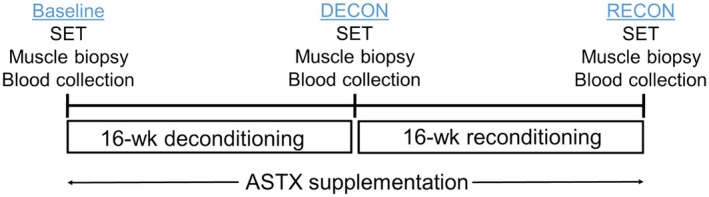
Timeline and experimental design. Horses underwent a 32‐week diet intervention, where they were assigned to a control group (CON; *n* = 6) or a treatment group which received astaxanthin supplementation (ASTX; *n* = 6). Horses participated in a 16‐week deconditioning program (DECON), followed by a 16‐week reconditioning period (RECON). Submaximal exercise tests (SET) were performed at the beginning of the study (Baseline), after DECON, and after RECON. Muscle biopsy samples were collected from the middle section of semitendinosus 2 weeks prior to and 2 h after each SET. Samples were used for analyses of oxidative status (superoxide dismutase; SOD, glutathione peroxidase; GPX, and malondialdehyde; MDA), mitochondrial respirometry, and mRNA gene expression. Blood samples were collected 30 min before and 0, 15, 30, and 60 min after each SET and used for analyses of oxidative status (SOD, GPX, MDA, protein carbonyl, and 8‐hydroxy‐2′‐deoxyguanosine).

Prior to the study, all horses were actively participating in an exercise program consisting of 60 min of polo lessons 4 days per week for at least 6 months. At the start of the study, horses began a 16 weeks deconditioning program (DECON, Figure 1). During this period, horses received turnout approximately 4 h per days, 5 days per week in the same paddock, with no forced exercise. After DECON, horses underwent a 16 weeks reconditioning period (RECON) during which time they received turnout approximately 4 h per day, 5 days per week and completed a progressive exercise training program 4 days per week (Table S1). The reconditioning program and submaximal exercise tests were completed by the same riders.

### 2.3 Diet treatment

Horses were blocked by BW, age, and sex and were randomly assigned to either a control group, which received no dietary supplement (CON; *n* = 6) or a treatment group supplemented with a commercially available astaxanthin supplement (ASTX; *n* = 6; Algalíf Iceland ehf., Reykjanesbaer, Iceland) that provided 75 mg of ASTX daily orally. The ASTX supplement contained NLT 10% astaxanthin oleoresin extracted from *Haematococcus pluvialis*, NMT 0.8% D‐α tocopherol (as a preservative), and NMT 0.2% rosemary extract in high oleic sunflower oil and fatty acids from *H. pluvialis*. The supplementation period started a week after the baseline submaximal exercise test (SET) and lasted for 32 weeks, during DECON and RECON. Astaxanthin was mixed with 15 g ProElite Senior concentrate (Cargill Inc., Wayzata, MN) to increase palatability. Control horses received 15 g ProElite Senior concentrate (Cargill Inc.) without ASTX.

Horses were individually fed twice daily with 0.3% BW/d ProElite Senior concentrate (Cargill Inc.) and 2% BW/d timothy grass mix hay in addition to the 15 g concentrate used to provide the supplement or as a control. The basal diet was formulated to meet the requirements for mature horses (NRC, 2007). Horses were provided with ad libitum access to a mineral salt block and water. Composited hay and grain samples were analyzed for nutrient composition at the Dairy One Forage Laboratory (Ithaca, NY) using standard wet‐chemistry procedures (Table S2).

### 2.4 Submaximal exercise tests

Horses underwent a 26 min ridden submaximal exercise test (SET) at the beginning of the study (Baseline), after DECON, and after RECON. The same group of riders was used during RECON and the SETs. All horses completed the SET on the same day. Heart rate (HR) was recorded using an equine heart rate monitor (Polar Equine, Polar Electro Inc., Woodbury, NY). Heart rate was averaged using all values within each step of the SET. Any lapses in measurement (“0” for heart rate) were discarded to not falsely reduce heart rate. SETs were designed to mimic a chukker, a playing period of a polo game. Each SET consisted of 5 min walk (1.83 ± 0.14 m/s) and 6 replicates of 2.5 min canter (4.64 ± 0.14 m/s), 30 s extended canter (5.88 ± 0.14 m/s), and 30 s walk (1.83 ± 0.14 m/s) followed by a 30 min walking recovery period (1.83 ± 0.14 m/s). To determine the velocity, the number of laps per horse was counted at each gait, where one lap consisted of 179.8 m. Then the travel distance was divided by the time (in second) taken. The velocity of each gait was not affected by the deconditioning‐reconditioning cycle (*p* = 0.13) or treatment (*p =* 0.61).

### 2.5 Sample collection and processing

Blood samples (approximately 20 mL) were collected 30 min before and 0, 15, 30, and 60 min after each SET. Samples were divided into tubes containing heparin or EDTA for plasma or containing no anticoagulant for separation of serum (Greiner Bio‐One, Monroe, NC). Plasma tubes were inverted 6 to 8 times after collection, kept on ice, and processed within 3 h by centrifugation at 3000*g* for 30 min at 4°C (Eppendorf Centrifuge 5810R, Framingham, MA). Serum tubes were kept at room temperature for 4–6 h to allow blood to clot and were stored at 4°C overnight and centrifuged the following morning. Plasma and serum were stored at −20°C until analysis.

Muscle biopsies were collected from the middle section of semitendinosus (STN) 2 weeks prior to (Pre‐Ex) and 2 h after (Post‐Ex) each SET. Biopsies were performed using a 14‐gauge SuperCore Semi‐Automatic Biopsy Instrument (Argon Medical Devices, Plano, TX) under sedation with dormosedan (Zoetis, Troy Hills, NJ). Approximately 10 cm^2^ of skin was shaved and cleansed with chlorhexidine and 70% ethanol solution before a local block with lidocaine (VET ONE, Boise, Idaho) was applied. A small incision was made through the skin, and the tissue collection needle was inserted to a depth of 4.0 cm. The biopsy site alternated between the right and left STN muscle at each biopsy time point. Samples for oxidative and gene expression analyses were immediately snap frozen in liquid nitrogen and were stored at −80°C until further use. Samples for high‐resolution respirometry were only collected pre‐exercise and were placed in ice cold BIOPS (2.77 mM CaK_2_EGTA, 7.23 mM K_2_EGTA, 50 mM K^+^ MES, 6.56 mM MgCl_2_, 20 mM Taurine, 5.77 mM ATP, 15 mM PCr, 0.5 mM DTT, and 20 mM Imidazole) and were processed within 10 h.

### 2.6 Oxidative stress markers

Serum was analyzed for creatine kinase (CK) activity at the University of Missouri Veterinary Medical Diagnostic Laboratory (Columbia, MO). Commercially available kits were used to analyze plasma protein carbonyl concentration (cat# 10005020; Cayman Chemical Company, Ann Arbor, MI) and serum 8‐OHdG concentration (cat# STA‐320; Cell Biolabs, Inc., San Diego, CA) according to the manufacturer's protocol. Plasma and muscle MDA concentrations were analyzed using a commercial assay kit (cat# NWK‐MDA01; Northwest Science Specialties LLC, Vancouver, WA) which was adapted for microplate reader quantification. Up to 10 mg of semitendinosus muscle biopsy samples were homogenized using TissueLyser II (QIAGEN, Germantown, MD) at 30 Hz for 3 min in a 10% w/v assay buffer provided in the kits. Samples were centrifuged at 11,000*g* for 3 min at 4°C. Supernatant was collected and used for the assay without further dilution. Plasma protein carbonyl and serum 8‐OHdG concentrations were analyzed in duplicate with the intra‐assay CV of 2.33% and 4.25%, respectively. Plasma and muscle MDA were analyzed in duplicate with the intra‐assay CV of 2.70% and 4.25%, respectively.

### 2.7 Antioxidant activity

Plasma and skeletal muscle were analyzed for SOD and GPX activities using commercially available kits (cat# 706002; cat# 7031012; Cayman Chemical Company) according to the manufacturer's protocol. Plasma was diluted 1:5 with sample buffer for the SOD activity assay and 1:2 for the GPX activity assay. To determine muscle SOD activity, up to 10 mg of muscle biopsy samples were homogenized using TissueLyser II (QIAGEN) at 30 Hz for 3 min in a 10% w/v buffer containing 1 mM EGTA (Sigma‐Aldrich), 210 mM mannitol (Alfa Aesar, Haverhill, MA), and 70 mM sucrose (Sigma‐Aldrich), followed by 5 min centrifugation at 1500*g* at 4°C. Supernatant was collected and diluted 1:50 with the assay buffer. To analyze muscle GPX activity, 10 mg of tissue was homogenized as previously described in a 10% w/v buffer with 50 mM Tris–HCL (Sigma‐Aldrich), 5 mM EDTA (Sigma‐Aldrich), and 1 mM DTT (Sigma‐Aldrich), followed by 15 min centrifugation at 10,000*g* at 4°C. Supernatant was collected and used undiluted. Plasma and muscle SOD activity were analyzed in duplicate with an intra‐assay CV of 4.54% and 4.34%, respectively. Plasma and muscle GPX activities were also analyzed in duplicate with an intra‐assay CV of 1.77% and 4.87%, respectively. Means of duplicate samples were used for statistical analysis.

### 2.8 Mitochondrial production of reactive oxygen species

Mitochondrial hydrogen peroxide (H_2_O_2_) concentration in permeabilized muscle fibers was measured using Amplex Red (Life Technologies, Carlsbad, CA) according to the manufacturer's protocol. Details of this assay have been previously outlined (Min et al., 2011, 2015). In essence, the assay relies on the ability of horseradish peroxidase (HRP) to catalyze the H_2_O_2_‐dependent oxidation of nonfluorescent Amplex Red, resulting in the production of fluorescent resorufin red. To convert all superoxide into H_2_O_2_, superoxide dismutase was added to the preparation. While the assay quantifies all H_2_O_2_ generated in the fiber, existing research suggests that the primary source of ROS production in the permeabilized muscle fiber preparation is mitochondria within the skeletal muscle fibers (Anderson et al., 2009; Anderson & Neufer, 2006). The measurement of mitochondrial ROS production was measured during state 3 respiration using the creatine kinase energy clamp technique to sustain respiration at a steady state using methods described previously (Min et al., 2015; Talbert et al., 2013).

### 2.9 High‐resolution respirometry

Muscle mitochondrial respiration was examined using the high‐resolution respirometer Oroboros O2k (OROBOROS Instruments, Innsbruck, Austria) following the methods previously reported (Kwon et al., 2016; Park et al., 2018). Briefly, fiber bundles in BIOPS were separated with sharp forceps and permeabilized in a BIOPS‐based saponin solution (50 μg saponin per mL BIOPS) for 30 min, followed by two 10 min rinses with ice cold mitochondrial respiration fluid (MIR05: 110 mM Sucrose, 0.5 mM EGTA, 3 mM MgCl_2_, 60 mM K‐lactobionate, 20 mM taurine, 10 mM KH_2_PO_4_, 20 mM HEPES, 1 g/L BSA; pH 7.1). After removal of excess liquid with filter paper, the wet mass of each sample was recorded. The sample was then transferred into a chamber (Oxytherm, Hansatech Instruments, UK) containing 2 mL MIR05 solution warmed to 37°C and allowed to equilibrate for 5 min. State 2 respiration was assessed with 10 mM glutamate (G5889, Sigma‐Aldrich) and 2 mM malate (M1000, Sigma‐Aldrich). With the addition of 5 mM ADP (A5285, Sigma‐Aldrich), complex I state 3 respiration (OXPHOS, Comp. I) was measured. Next, 10 mM succinate (S2378, Sigma‐Aldrich) was added to assess complex I and II state 3 respiration (OXPHOS, Comp. I&II), and 10 mM cytochrome c (C2506, Sigma‐Aldrich) was used to test membrane integrity. Finally, complex II state 3 (OXPHOS, Comp. II) respiration was determined with 0.5 μM rotenone (R8875, Sigma‐Aldrich). State 4 respiration (LEAK) was assessed by inhibiting complex V with 2 μM oligomycin (O4876, Sigma‐Aldrich). Samples with more than a 10% increase in respiration in response to cytochrome c were considered to have impaired membrane integrity and were excluded from the analysis. In each condition, the respiration rate was recorded for at least 3 min until a plateau was reached, and the average of the last minute was used for data analysis. Respiration data are presented relative to wet tissue weight (integrative). Due to limited sample availability, technical replicates were not performed for each sample in this study. No muscle fibers were excluded from any samples during the experimental procedure.

### 2.10 Real‐time reverse transcription polymerase chain reaction (RT‐PCR)

Tissue homogenization, RNA extraction, and genomic DNA removal were performed using RNeasy Fibrous Mini Kit (74704; QIAGEN) according to the manufacturer's protocol. The quality of RNA was determined using the TapeStation system (Agilent, Santa Clara, CA). Extracted RNA (10 μL) was reverse transcribed with M‐MLV (Invitrogen, Waltham, MA) according to the manufacturer's protocol. Real‐time RT‐PCR primers were designed using NCBI Primer‐BLAST, synthesized by Integrated DNA Technologies (Coralville, IA), and validated as previously described (Govoni et al., 2006). Real‐time RT‐PCR was performed in triplicate using Power SybrGreen Master Mix (A25742; Applied Biosystems, Waltham, MA) and the QuantStudio 6 Pro Real‐Time PCR System (Applied Biosystems, Waltham, MA). Each reaction contained 5.0 μL cDNA, 3.0 μL nuclease‐free water, 1.0 μL each forward and reverse primer at 10 nmol/L (Table S3), and 10 μL SybrGreen, resting in a total reaction volume of 20 μL. ^Δ^Ct values were determined and used for ^ΔΔ^Ct value calculation to obtain relative gene expression (Livak & Schmittgen, 2001). Means of triplicate samples were used for statistical analysis. Tubulin alpha‐1A chain (TUBA1) mRNA expression was used as an endogenous control and was not different between treatment groups (*p* ≥ 0.20).

### 2.11 Statistical analysis

Data were properly formatted and analyzed using the R programming language in the R studio (version 4.2.1; R Core Team, 2022) on “Spotted Wakerobin” release for macOS, using the packages car (Fox & Weisberg, 2018), emmeans (Lenth, 2023), lme4 (Bates et al., 2015), performance (Lüdecke et al., 2021), and tidyverse (Wickham et al., 2019). Outcomes were analyzed using linear mixed effects models. For a comparison between DECON and RECON, data for heart rate, and circulating and muscle oxidative status factors were analyzed for fixed effects of diet (CON and ASTX), conditioning status (after DECON and after RECON), and time point relative to the exercise test (before [Pre‐Ex] and after [Post‐Ex] SET) and all the interactions. In the circulating factor analysis, no statistical difference was identified between 0, 15, 30, and 60 min after SET, so the mean for each variable was calculated and used as a Post‐Ex value (*p* ≥ 0.15). Data for muscle mitochondrial variables and gene expression were tested for fixed effects of diet and conditioning status and the interaction. Values at Baseline were included as a fixed effect covariate as ASTX supplementation started after the first SET. Sex, age, and BW were also treated as a fixed effect covariate. For a comparison between Baseline and DECON as well as Baseline and RECON, a paired sample *t*‐test was performed on SET. Then, a difference between the SETs was determined and included in a linear regression model to assess the fixed effects of diet, where sex, age, and BW were treated as a fixed effect covariate. All data are expressed as estimated marginal (least squared) mean ± SEM. Statistical significance was considered at *p* ≤ 0.05.

## 3 RESULTS

### 3.1 Astaxanthin supplementation and training status do not impact HR during SET and a recovery period

Average HR during exercise was not affected by ASTX (*p =* 0.22; Table 1) or a deconditioning‐reconditioning cycle (*p =* 0.32). There was no effect of ASTX or conditioning status on average recovery HR (*p* ≥ 0.28).

**TABLE 1:** Gait velocity^1^ and heart rate (HR)^2^ during standardized exercise tests^3^.

	Baseline		DECON		RECON	
	CON	ASTX	CON	ASTX	CON	ASTX
Velocity						
Walk	1.9 ± 0.3	1.5 ± 0.3	2.3 ± 0.3	2.3 ± 0.3	1.6 ± 0.4	1.5 ± 0.3
Canter	4.9 ± 0.3	4.6 ± 0.3	4.6 ± 0.3	4.8 ± 0.3	4.3 ± 0.4	4.7 ± 0.3
Extended canter	5.8 ± 0.3	5.7 ± 0.3	5.5 ± 0.3	6.2 ± 0.3	5.9 ± 0.4	6.3 ± 0.3
HR						
Walk	127 ± 5	139 ± 5	129 ± 5	135 ± 5	143 ± 5	141 ± 5
Canter	115 ± 5	123 ± 5	128 ± 5	134 ± 5	135 ± 5	127 ± 5
Extended canter	124 ± 5	137 ± 5	132 ± 5	141 ± 5	148 ± 5	133 ± 5
Recovery	64.1 ± 2.6	67.9 ± 3.2	64.7 ± 3.7	71.6 ± 3.8	65.7 ± 3.9	67.3 ± 3.8

^1^ m/s; lsmean ± SEM.

^2^ beats/min; lsmean ± SEM.

^3^ Baseline = before deconditioning; DECON = after deconditioning; RECON = after reconditioning. *N* = 6 horses per diet at Baseline and DECON, *n* = 5 CON and *n* = 6 ASTX horses at RECON. The velocity of each gait was not affected by the deconditioning‐reconditioning cycle (*p* = 0.13) or treatment (*p =* 0.61). There were no effects of ASTX (*p =* 0.22) or a deconditioning–reconditioning cycle (*p =* 0.32) on HR. There was no effect of ASTX or conditioning status on average recovery HR (*p* ≥ 0.28).

### 3.2 Astaxanthin supplementation and reconditioning increase circulating antioxidant activity

**FIGURE 2:**
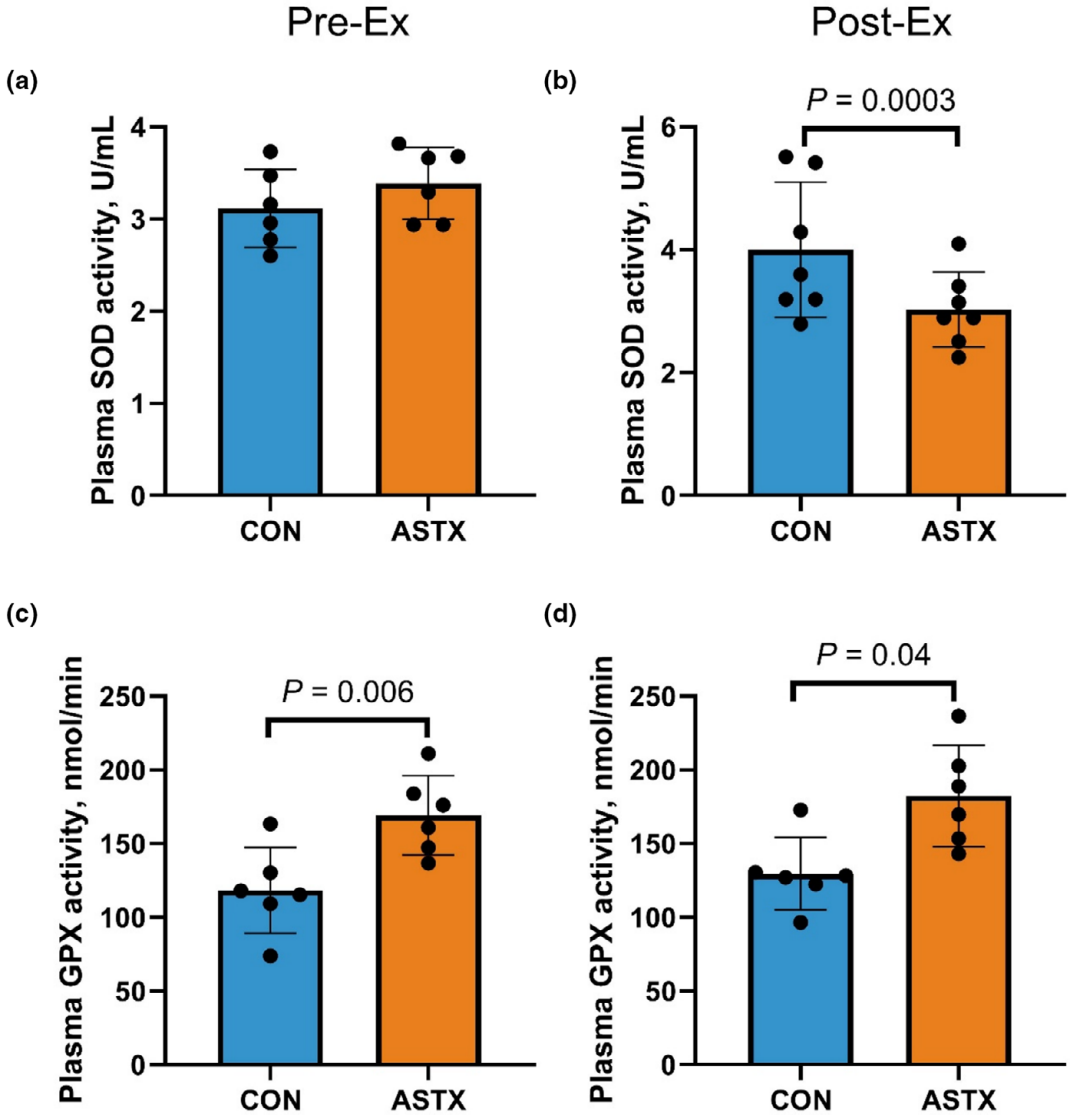
Effects of astaxanthin supplementation on antioxidant activities in circulation (*n* = 12). Superoxide dismutase (SOD; a and b) and glutathione peroxidase (GPX; c and d) activities were measured in plasma of control (CON; *n* = 6 horses) and astaxanthin (ASTX; *n* = 6 horses) supplemented horses before (Pre‐Ex; a and c) and after submaximal exercise tests (Post‐Ex; b and d). A linear mixed effect model was used to determine the effects of diet (CON and ASTX) where Baseline was treated as a fixed effect covariate.

There was no observed effect of ASTX supplementation on plasma SOD activity at Pre‐Ex (*p* = 0.27; Figure 2a). Superoxide dismutase activity at Post‐Ex was affected by ASTX, where SOD was greater in CON than ASTX regardless of DECON and RECON (*p* = 0.0003; Figure 2b). There was a main treatment effect on plasma GPX activity, where ASTX had greater GPX activity than CON at Pre‐ (*p* = 0.006; Figure 2c) and Post‐Ex (*p* = 0.04; Figure 2d).

Circulating antioxidant activity was also altered by a deconditioning‐reconditioning cycle. Deconditioning did not affect SOD activity at Pre‐Ex (*p* = 0.11; Table 2). Following RECON, Pre‐Ex SOD activity was greater than DECON (*p* = 0.004) but was not different from Baseline (*p* = 0.34). Within Post‐Ex, SOD activity remained unchanged from Baseline to DECON (*p* = 0.23) while there was an increase at RECON relative to DECON (*p* = 0.002). There was no observed difference between Baseline and RECON (*p* = 0.08) in SOD activity. Deconditioning did not affect GPX activity at Pre‐ or Post‐Ex (*p* ≥ 0.81; Table 2). Following RECON, Pre‐ and Post‐Ex GPX activity was greater than Baseline and DECON (*p* ≤ 0.002).

**TABLE 2:** Antioxidant activities in circulation at rest (Pre‐Ex) and after submaximal exercise test (Post‐Ex).

Item^1^	Baseline^2^	DECON	RECON
SOD^3^ activity, U/mL			
Pre‐Ex	3.39 ± 0.29^abx^	2.80 ± 0.20^ax^	3.78 ± 0.15^bx^
Post‐Ex	3.52 ± 0.30^abx^	3.08 ± 0.19^ay^	4.13 ± 0.14^by^
GPX^4^ activity, nmol/min/mL			
Pre‐Ex	125.88 ± 10.97^ax^	123.88 ± 12.53^ax^	177.89 ± 12.16^bx^
Post‐Ex	144.09 ± 12.11^ay^	139.87 ± 12.45^ay^	186.17 ± 12.21^by^

*Note*: Within Pre‐Ex or Post‐Ex, means with different superscript letters (a, b) differ (*p* ≤ 0.05). A linear mixed effect model was used to determine the effects of conditioning status (DECON and after RECON) where Baseline was treated as a fixed effect covariate. A paired sample *t*‐test was performed on SET for a comparison between Baseline and DECON, and Baseline and RECON. Within time point, means with different superscript letters (x, y) differ (*p* ≤ 0.05). A paired sample *t*‐test was performed on time point at Baseline for a comparison between Pre‐ and Post‐Ex. A linear mixed effects model was used to determine the effects of time point (Pre‐Ex and Post‐Ex) at DECON and RECON, where Baseline was treated as a fixed effect covariate.

^1^ Mean ± SEM.

^2^ Baseline = before deconditioning (*n* = 12 horses); DECON = after deconditioning (*n* = 12 horses); RECON = after reconditioning (*n* = 11 horses).

^3^ Superoxide dismutase.

^4^ Glutathione peroxidase.

At Baseline, SOD activity did not differ between Pre‐ and Post‐Ex (*p* = 0.21; Table 2). Following DECON and RECON, SOD activity was increased in response to SET (*p* ≤ 0.04). Additionally, GPX activity was greater at Post‐Ex than at Pre‐Ex at Baseline, DECON, and RECON (*p* ≤ 0.02).

### 3.3 Astaxanthin supplementation during reconditioning mitigates circulating protein damage

**FIGURE 3:**
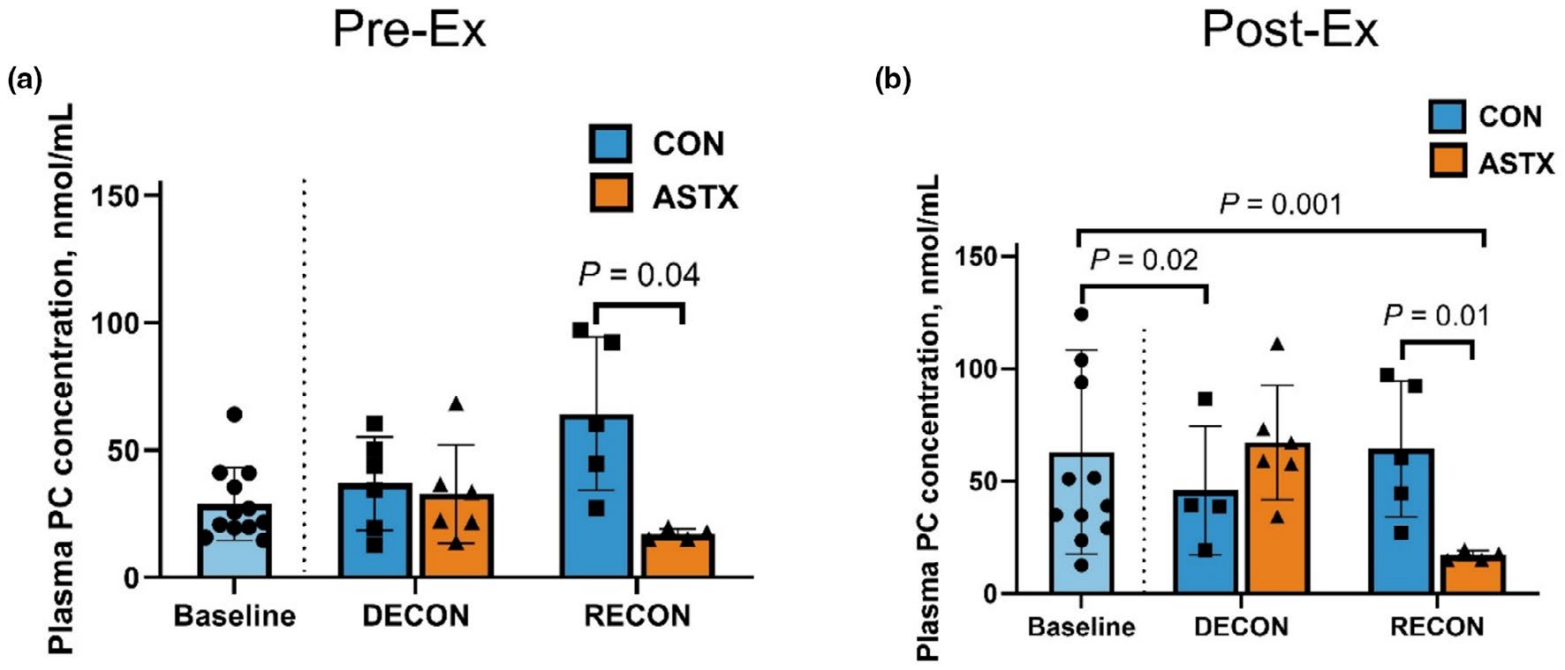
Effects of astaxanthin supplementation on protein oxidative damage in circulation (*n* = 12). Protein carbonyl concentration was measured in plasma of control (CON; *n* = 6 horses at Baseline and DECON, *n* = 5 horses at RECON) and astaxanthin (ASTX; *n* = 6 horses at Baseline and DECON, *n* = 5 horses at RECON) supplemented horses before exercise (Pre‐Ex; a) and after submaximal exercise tests (Post‐Ex; b). Linear mixed effect model was used to determine the effects of diet (CON and ASTX) and conditioning status (DECON and RECON) and the interaction where Baseline was treated as a fixed effect covariate. A difference between Baseline and DECON as well as Baseline and RECON was determined and included in a linear regression model to assess the fixed effects of diet.

An interaction of ASTX and a deconditioning‐reconditioning cycle was observed in Pre‐ and Post‐Ex plasma protein carbonyl concentration (*p* ≤ 0.05). Protein carbonyl concentration at Pre‐Ex did not differ between CON and ASTX at DECON (*p* = 0.86; Figure 3a), yet ASTX had less protein carbonylation than CON at RECON (*p* = 0.04). Supplementation of ASTX did not affect changes in Pre‐Ex protein carbonyl concentration from Baseline to DECON or from Baseline to RECON (*p* ≥ 0.25). At Post‐Ex, horses supplemented with ASTX had less protein carbonylation than CON after RECON (*p* = 0.01; Figure 3b). Protein carbonyl concentration at Post‐Ex decreased in CON from Baseline to DECON (*p* = 0.02) yet was not different in CON between Baseline and RECON (*p* = 0.36). In ASTX, there was a decrease in protein carbonyl concentration at RECON compared with Baseline (*p* = 0.001).

There was no observed effect of ASTX supplementation on plasma MDA concentration (*p* = 0.33), serum 8‐OHdG concentration (*p* = 0.92), or serum CK activity (*p* = 0.63), which are biomarkers of lipid, DNA, and muscle damage, respectively.

A deconditioning‐reconditioning cycle impacted oxidative stress markers in circulation. Following DECON, MDA concentration was greater than Baseline at both Pre‐ and Post‐Ex (*p* ≤ 0.005; Table 3). After RECON, MDA concentration was similar to Baseline at Pre‐ and Post‐Ex (*p* = 0.28). The concentration of 8‐OHdG following DECON was greater at Pre‐ and Post‐Ex than Baseline (*p* ≤ 0.02). Plasma 8‐OHdG at Pre‐ and Post‐Ex was not different between Baseline and RECON (*p* ≥ 0.89). Deconditioning increased Pre‐ and Post‐Ex CK activity compared with Baseline (*p* ≤ 0.05). Following RECON, Pre‐Ex CK activity was greater than Baseline (*p* = 0.01) and was not different from DECON (*p* = 0.45). However, Post‐Ex CK activity was not impacted by RECON compared with Baseline and DECON (*p* ≥ 0.10).

**TABLE 3:** Oxidative stress and muscle damage markers at rest (Pre‐Ex) and after submaximal exercise test (Post‐Ex).

Item^1^	Baseline^2^	DECON	RECON
MDA^3^ concentration, nmol/mL			
Pre‐Ex	0.50 ± 0.03^ax^	0.70 ± 0.05^bx^	0.58 ± 0.08^abx^
Post‐Ex	0.61 ± 0.02^ay^	0.82 ± 0.06^by^	0.61 ± 0.05^ax^
Protein carbonyl concentration, nmol/mL			
Pre‐Ex	28.83 ± 4.14^ax^	34.77 ± 5.73^ax^	43.23 ± 9.92^ax^
Post‐Ex	67.04 ± 16.10^ay^	58.61 ± 8.67^ay^	55.91 ± 11.35^ax^
8‐OHdG^4^ concentration, ng/mL			
Pre‐Ex	17.28 ± 1.78^ax^	21.86 ± 1.67^bx^	16.60 ± 2.80^abx^
Post‐Ex	17.84 ± 1.63^ax^	23.00 ± 1.68^bx^	17.54 ± 2.98^abx^
CK^5^ activity, U/L			
Pre‐Ex	139.33 ± 11.10^ax^	174.58 ± 15.10^bx^	194.64 ± 21.34^bx^
Post‐Ex	183.04 ± 22.89^ay^	222.58 ± 17.47^by^	278.41 ± 76.32^abx^

*Note*: Within Pre‐Ex or Post‐Ex, means with different superscript letters (a, b) differ (*p* ≤ 0.05). A linear mixed effect model was used to determine the effects of conditioning status (DECON and RECON) where Baseline was treated as a fixed effect covariate. A paired sample *t*‐test was performed on SET for a comparison between Baseline and DECON, and Baseline and RECON. Within time point, means with different superscript letters (x, y) differ (*p* ≤ 0.05). A paired sample *t*‐test was performed on time point at Baseline for a comparison between Pre‐ and Post‐Ex. A linear mixed effect model was used to determine the effects of time point (Pre‐Ex and Post‐Ex) at DECON and RECON, where Baseline was treated as a fixed effect covariate.

^1^ Mean ± SEM.

^2^ Baseline before deconditioning (*n* = 12 horses); DECON = after deconditioning (*n* = 12 horses); RECON = after reconditioning (*n* = 11 horses).

^3^ Malondialdehyde.

^4^ 8‐hydroxy‐2′‐deoxyguanosine.

^5^ Creatine kinase.

Submaximal exercise test affected some of the circulating oxidative stress markers. At Baseline and DECON, Post‐Ex MDA concentration was greater than Pre‐Ex (*p* ≤ 0.05; Table 3), yet it remained unchanged at RECON (*p* = 0.26). In response to SET, there was an increase in protein carbonyl concentration at Baseline (*p* = 0.05; Table 3) and DECON (*p* = 0.008), which was not observed at RECON (*p* = 0.26). There was no effect of SET on 8‐OHdG concentration at Baseline, DECON, or RECON (*p* ≥ 0.17; Table 3). At Baseline and DECON, CK activity was greater at Post‐Ex than Pre‐Ex (*p* ≤ 0.04; Table 3). Following RECON, there was no observed effect of SET on CK activity (*p* = 0.21).

### 3.4 Training status, but not ASTX supplementation, improves oxidative status in muscle

In the STN muscle, there were no main effects of ASTX at any time point on SOD (*p* = 0.98), GPX (*p* = 0.51), or MDA (*p* = 0.21). However, an interaction of conditioning status and time point relative to SET was observed. Within Pre‐Ex, DECON and RECON did not alter muscle SOD activity (*p* ≥ 0.30; Table 4). Within Post‐Ex, SOD activity increased following DECON relative to Baseline and returned to baseline following RECON (*p* ≥ 0.05). Within Pre‐Ex, muscle GPX decreased at DECON relative to Baseline (*p* = 0.004; Table 4) and increased at RECON from DECON (*p* = 0.05). Within Post‐Ex, GPX activity decreased at RECON compared with Baseline (*p* = 0.01). Muscle MDA concentration at Pre‐Ex increased at DECON and RECON relative to Baseline (*p* ≤ 0.05; Table 4). Pre‐Ex MDA was not different between DECON and RECON (*p* = 0.66). There was no observed effect of DECON on Post‐Ex MDA concentration relative to Baseline (*p* = 0.07). Reconditioning decreased Post‐Ex MDA compared with Baseline (*p* = 0.01), but there was no difference between DECON and RECON (*p* = 0.55).

**TABLE 4:** Antioxidant activities and oxidative stress markers in semitendinosus muscle at rest (Pre‐Ex) and after submaximal exercise test (Post‐Ex).

Item^1^	Baseline^2^	DECON	RECON
SOD^3^ activity, U/mg			
Pre‐Ex	137.97 ± 4.76^ax^	135.20 ± 3.21^ax^	142.33 ± 4.90^ax^
Post‐Ex	144.93 ± 3.73^ax^	161.77 ± 6.06^by^	145.75 ± 5.62^ax^
GPX^4^ activity, nmol/min/mg			
Pre‐Ex	108.54 ± 4.10^ax^	89.61 ± 3.80^bx^	109.03 ± 6.26^ax^
Post‐Ex	134.84 ± 6.50^ay^	128.98 ± 7.19^aby^	115.82 ± 6.40^bx^
MDA^5^ concentration, nmol/mg			
Pre‐Ex	1.17 ± 0.11^ax^	1.82 ± 0.28^bx^	1.82 ± 0.24^bx^
Post‐Ex	1.81 ± 0.18^ax^	1.33 ± 0.20^abx^	1.09 ± 0.26^bx^

*Note*: Within Pre‐Ex or Post‐Ex, means with different superscript letters (a, b) differ (*p* ≤ 0.05). A linear mixed effect model was used to determine the effects of conditioning status (DECON and RECON) where Baseline was treated as a fixed effect covariate. A paired‐sample *t*‐test was performed on SET for a comparison between Baseline and DECON, and Baseline and RECON. Within time point, means with different superscript letters (x, y) differ (*p* ≤ 0.05). A paired sample *t*‐test was performed on time point at Baseline for a comparison between Pre‐ and Post‐Ex. A linear mixed effect model was used to determine the effects of time point (Pre‐Ex and Post‐Ex) at DECON and RECON, where Baseline was treated as a fixed effect covariate.

^1^ Mean ± SEM.

^2^ Baseline = before deconditioning (*n* = 12 horses); DECON = after deconditioning (*n* = 12 horses); RECON = after reconditioning (*n* = 11 horses).

^3^ Superoxide dismutase.

^4^ Glutathione peroxidase.

^5^ Malondialdehyde.

In response to SET, an increase in Post‐Ex SOD activity was observed at DECON (*p* < 0.001; Table 4) whereas there was no observed difference between Pre‐ and Post‐Ex at Baseline or RECON (*p* ≥ 0.21). At Baseline and after DECON, there was an increase in GPX activity following SET (*p* ≤ 0.002). However, GPX activity was similar between Pre‐ and Post‐Ex after RECON (*p* = 0.21). There was no observed effect of exercise test on muscle MDA concentration (*p* ≥ 0.06) at any time point.

### 3.5 Reconditioning, but not ASTX supplementation, improves deconditioning‐induced increase in ROS and decrease in mitochondrial respiration

**FIGURE 4:**
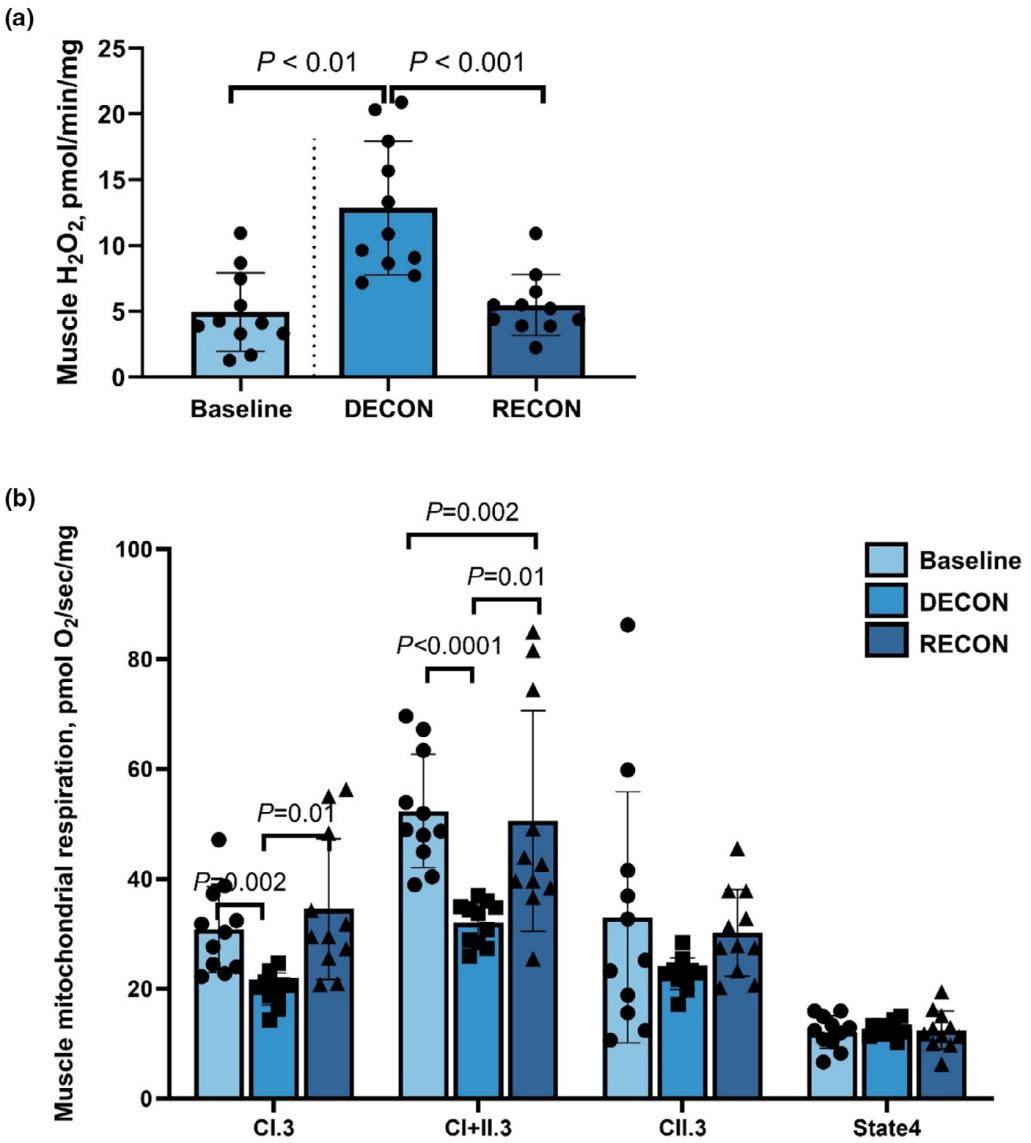
Effects of deconditioning (DECON; *n* = 12 horses) and reconditioning (RECON; *n* = 11 horses) periods on reactive oxygen species production (H_2_O_2_: a) and mitochondrial respiration normalized to tissue wet weight (integrative; b) in skeletal muscle at rest. Linear mixed effect model was used to determine the effects of conditioning status (DECON and RECON) where Baseline was treated as a fixed effect covariate. A paired sample *t*‐test was performed on SET for a comparison between Baseline and DECON, and Baseline and RECON. A difference between Baseline and DECON as well as Baseline and RECON was determined and included in a linear regression model to assess the fixed effects of diet.

Only DECON and RECON affected ROS emission. There was an increase in H_2_O_2_ concentration from Baseline to DECON (*p* < 0.001; Figure 4a), and a decrease from DECON to RECON (*p* < 0.001). Muscle H_2_O_2_ was not different between Baseline and RECON (*p* = 0.83).

There was no observed effect of ASTX on muscle integrative mitochondrial respiratory capacity. In contrast, OXPHOS, Comp. I respiration decreased following DECON compared with Baseline (*p* = 0.002; Figure 4b). Following RECON, OXPHOS, Comp. I respiration was greater than DECON (*p* = 0.01) and was not different from Baseline (*p* = 0.79). Deconditioning reduced OXPHOS, Comp. I&II respiration compared with Baseline (*p* < 0.001). OXPHOS, Comp. I&II respiration at RECON was greater than DECON (*p* = 0.01) yet was less than Baseline (*p* = 0.002). OXPHOS, Comp. II respiration following DECON and RECON did not differ from Baseline (*p* ≥ 0.13). However, the respiration was greater at RECON than DECON (*p* = 0.02). A deconditioning‐reconditioning cycle did not affect LEAK respiration (*p* = 0.58).

### 3.6 Astaxanthin supplementation and reconditioning upregulate gene expression related to mitochondrial biogenesis and respiration in resting muscle

Supplementation of ASTX impacted gene expression related to the mitochondrial electron transport chain (ETC) in resting (Pre‐Ex) muscle, regardless of conditioning status (Table 5). Expression of ATP5F1D, a regulator of complex IV, was upregulated in ASTX relative to CON (*p* = 0.05). Horses supplemented with ASTX had greater expression of MDH1 and MDH2, which enhance NADH transportation from the cytoplasm into mitochondria, than CON (*p* ≤ 0.03). Expression of SDHB and SDHC, which regulate complex II function, was upregulated in ASTX relative to CON across time points (*p* ≤ 0.05).

**TABLE 5:** Effects of astaxanthin (ASTX) supplementation on gene expression^1^ in Pre‐Ex^2^ semitendinosus muscle.

Item	CON	ASTX	*p* Value
ATP5F1D	1.06 ± 0.11^a^	1.47 ± 0.21^b^	0.05
MDH1	1.09 ± 0.12^a^	1.65 ± 0.29^b^	0.03
MDH2	1.05 ± 0.08^a^	1.53 ± 0.24^b^	0.04
SDHB	1.07 ± 0.10^a^	1.72 ± 0.25^b^	0.03
SDHC	1.08 ± 0.10^a^	1.69 ± 0.23^b^	0.05

*Note*: Means with different superscript letters differ (*p* ≤ 0.05). A linear mixed effect model was used to determine the effects of diet (CON, *n* = 5 horses and ASTX, *n* = 6 horses) where Baseline was treated as a fixed effect covariate. There was no main effect of conditioning status on the expression of these genes, so data were combined.

^1^ Relative to CON; mean ± SEM.

^2^ Muscle biopsies were performed 2 weeks before the submaximal exercise test (SET).

Gene expression related to mitochondrial biogenesis and respiration and redox homeostasis was altered by DECON and RECON in Pre‐Ex muscle (Table 6). Expression of genes involved in mitochondrial fusion, such as MFN1 and OPA1, was upregulated at DECON, respectively, compared with Baseline (*p* ≤ 0.008). The expression at RECON was greater than Baseline (*p* ≤ 0.01) but was not different from DECON (*p* ≥ 0.82). Muscle DNM1 expression, a mitochondrial fission regulator, was maintained between Baseline and DECON (*p* = 0.12). Following RECON, DNM1 was downregulated compared with Baseline (*p* = 0.005) but was not different from DECON (*p* = 0.23). The ETC complex related genes, including COX4I1, COX4I2, UCP2, and UQCRB, were greater at DECON than Baseline (*p* ≤ 0.02) but were similar between Baseline and RECON (*p* ≥ 0.07) and between DECON and RECON (*p* ≥ 0.22). Upregulation of NDFUA9, SDHA, SDHB, SDHC, and SDHD was also observed in response to DECON (*p* ≤ 0.01). Expression of these genes following RECON remained similar to DECON (*p* ≥ 0.15) but greater than Baseline (*p* ≤ 0.04). In a similar manner, expression of NFE2L2, which regulates antioxidant response, was greater at DECON than Baseline (*p* = 0.007) while at RECON, NFE2L2 was different only from Baseline (*p* = 0.01).

**TABLE 6:** Effects of deconditioning (DECON) and reconditioning (RECON) on gene expression^1^ in Pre‐Ex^2^ semitendinosus muscle.

Item	Baseline	DECON	RECON	*p* Value
ATP5F1D	1.19 ± 0.24^a^	2.07 ± 0.22^b^	1.71 ± 0.20^b^	0.01
COX4I1	1.10 ± 0.14^a^	1.88 ± 0.24^b^	1.29 ± 0.21^ab^	0.02
COX4I2	1.05 ± 0.10^a^	1.45 ± 0.11^b^	1.38 ± 0.20^ab^	0.01
DNM1	1.10 ± 0.13^a^	0.80 ± 0.13^ab^	0.60 ± 0.06^b^	0.01
MFN1	1.07 ± 0.06^a^	1.75 ± 0.12^b^	1.86 ± 0.39^b^	0.01
MFN2	1.02 ± 0.07^a^	1.94 ± 0.20^b^	2.12 ± 0.26^ab^	0.01
NDFUA9	1.08 ± 0.05^a^	2.17 ± 0.32^b^	1.95 ± 0.24^b^	0.03
NFE2L2	1.11 ± 0.16^a^	2.25 ± 0.29^b^	2.14 ± 0.34^b^	0.02
MDH1	1.14 ± 0.22^a^	2.59 ± 0.35^b^	1.98 ± 0.32^a^	< 0.01
MDH2	1.13 ± 0.19^a^	2.12 ± 0.30^b^	1.71 ± 0.20^b^	0.01
OPA1	0.94 ± 0.16^a^	2.40 ± 0.36^b^	2.12 ± 0.39^b^	0.01
SDHA	1.15 ± 0.21^a^	2.63 ± 0.27^b^	2.35 ± 0.32^b^	0.01
SDHB	1.11 ± 0.18^a^	2.71 ± 0.32^b^	1.64 ± 0.18^b^	0.01
SDHC	1.13 ± 0.21^a^	2.43 ± 0.29^b^	1.91 ± 0.27^b^	<0.01
SDHD	1.05 ± 0.04^a^	1.76 ± 0.31^b^	1.81 ± 0.39^b^	0.04
TXNRD2	1.09 ± 0.08^a^	1.31 ± 0.27^ab^	1.43 ± 0.11^b^	0.05
UCP2	1.11 ± 0.14^a^	1.57 ± 0.13^b^	1.48 ± 0.25^ab^	0.02
UCP3	1.07 ± 0.13	1.10 ± 0.16	1.55 ± 0.23	0.51
UQCRB	1.06 ± 0.05^a^	1.58 ± 0.18^b^	1.50 ± 0.15^ab^	0.01

*Note*: Means with different superscripts differ (*p* ≤ 0.05). Linear mixed effect model was used to determine the effects of conditioning status (DECON and RECON) where Baseline was treated as a fixed effect covariate. A paired sample *t*‐test was performed on SET for a comparison between Baseline and DECON, and Baseline and RECON. Baseline = before deconditioning (*n* = 12 horses); DECON = after deconditioning (*n* = 12 horses); RECON = after reconditioning (*n* = 11 horses).

^1^ Relative to Baseline; mean ± SEM.

^2^ Muscle biopsies were performed 2 weeks before the submaximal exercise test (SET).

**FIGURE 5:**
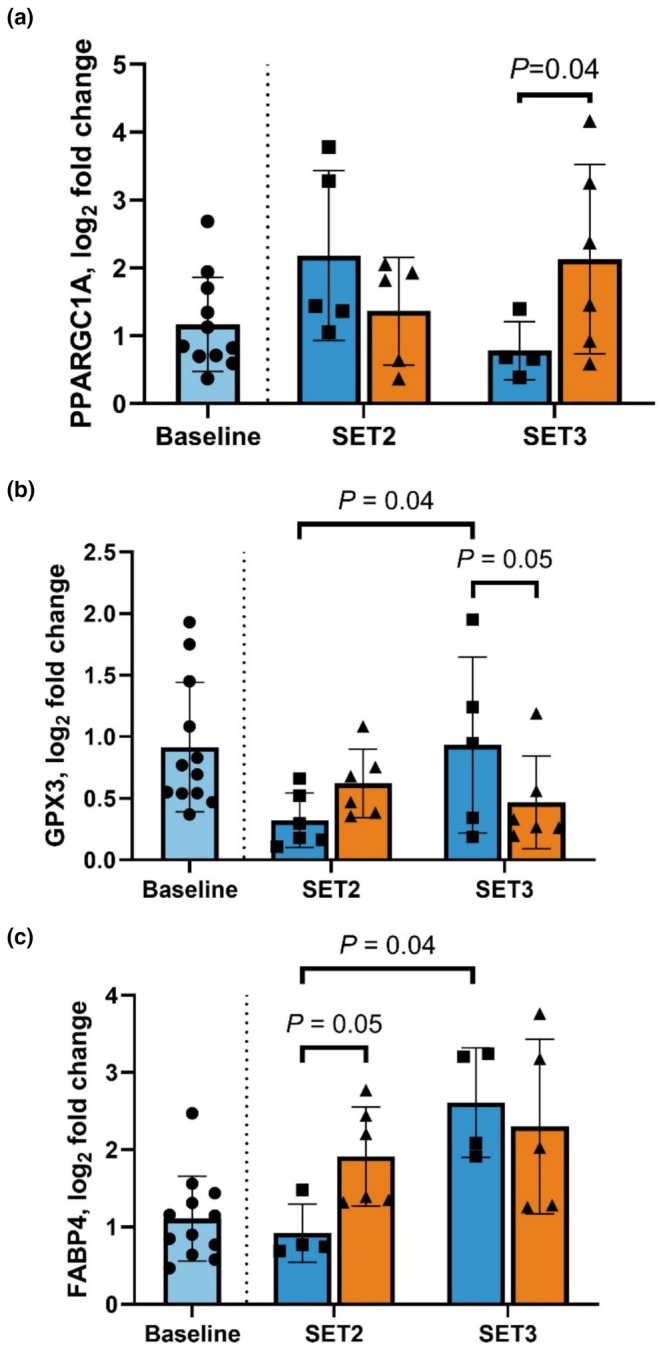
Effects of astaxanthin (ASTX) supplementation, deconditioning (DECON), and reconditioning (RECON) on gene expression in the semitendinosus (STN) muscle before (a) and after (b, c) exercise. All comparisons were made relative to Baseline. A linear mixed effect model was used to determine the effects of diet (CON; *n* = 6 horses and ASTX; *n* = 6 horses) and conditioning status (DECON and RECON) and the interaction where Baseline was treated as a fixed effect covariate. A difference between Baseline and DECON as well as Baseline and RECON was determined and included in a linear regression model to assess the fixed effects of diet.

There was an interaction of ASTX supplementation and a deconditioning‐reconditioning cycle on PPARGC1A, a master regulator of mitochondrial biogenesis (Figure 5a). Expression of PPARGC1A did not differ between CON and ASTX after DECON (*p* = 0.76). However, muscle PPARGC1A expression was greater in ASTX than CON after RECON (*p* = 0.02). Further, PPARGC1A in ASTX was greater at RECON than Baseline (*p* = 0.03).

### 3.7 A deconditioning‐reconditioning cycle has greater impacts on gene expression in exercising muscle than ASTX supplementation

Effects of DECON and RECON were observed on gene expression involved in mitochondrial biogenesis and respiration, redox state, and lipid metabolism in skeletal muscle after an SET (Table 7). Expression of FABP3 and MDH1 was greater at DECON and RECON than Baseline (*p* ≤ 0.05) but was not different between DECON and RECON (*p* ≥ 0.54). Similarly, COX4I2, PPARGC1, SOD1, and SOD2 expression was greater at RECON than Baseline (*p* ≤ 0.04) but remained unchanged between Baseline and DECON (*p* ≥ 0.08) and between DECON and RECON (*p* ≥ 0.38). Reconditioning increased MFN2 and SLC2A4 expression compared with Baseline (*p* ≤ 0.03). However, there was no observed effect of DECON relative to Baseline (*p* ≥ 0.09).

**TABLE 7:** Effects of deconditioning (DECON) and reconditioning (RECON) gene expression^1^ in Post‐Ex^2^ semitendinosus muscle.

Item	Baseline	DECON	RECON	*p* Value
COX4I1	1.21 ± 0.23	1.61 ± 0.23	1.59 ± 0.26	0.82
COX4I2	1.17 ± 0.21^a^	2.04 ± 0.39^ab^	1.68 ± 0.17^b^	0.04
CPT1A	1.20 ± 0.25	1.59 ± 0.29	1.71 ± 0.31	0.67
DNM1	1.09 ± 0.12	0.97 ± 0.18	0.78 ± 0.22	0.36
FABP3	1.13 ± 0.17^a^	2.01 ± 0.28^b^	2.41 ± 0.29^b^	0.02
MDH1	1.19 ± 0.22^a^	1.92 ± 0.39^b^	1.77 ± 0.30^b^	0.04
MDH2	1.16 ± 0.21	1.79 ± 0.30	1.66 ± 0.21	0.74
MFN1	1.17 ± 0.19	1.51 ± 0.35	1.65 ± 0.25	0.83
MFN2	1.10 ± 0.15^a^	1.88 ± 0.35^ab^	1.67 ± 0.23^b^	0.03
NEF2L2	1.43 ± 0.33	1.37 ± 0.30	1.40 ± 0.21	0.99
OPA1	1.17 ± 0.18	1.06 ± 0.21	1.26 ± 0.23	0.98
PPARγ	1.15 ± 0.20	1.54 ± 0.34	1.16 ± 0.18	0.84
PPARGC1A	1.12 ± 0.17^a^	1.50 ± 0.28^ab^	1.94 ± 0.26^b^	0.02
SLC2A4	1.16 ± 0.22^a^	1.55 ± 0.27^ab^	2.37 ± 0.28^b^	0.01
SOD1	1.14 ± 0.21^a^	1.62 ± 0.30^ab^	1.81 ± 0.27^b^	0.04
SOD2	1.23 ± 0.25^a^	1.38 ± 0.26^ab^	2.08 ± 0.27^b^	0.01

*Note*: Means with different superscript letters differ (*p* ≤ 0.05). A linear mixed effect model was used to determine the effects of conditioning status (DECON and RECON) where Baseline was treated as a fixed effect covariate. A paired‐sample *t*‐test was performed on SET for a comparison between Baseline and DECON, and Baseline and RECON. Baseline = before deconditioning (*n* = 12 horses); DECON = after deconditioning (*n* = 12 horses); RECON = after reconditioning (*n* = 11 horses).

^1^ Relative to Baseline; mean ± SEM.

^2^ Muscle biopsies were performed 2 h after submaximal exercise test (SET).

An interaction of ASTX supplementation and a deconditioning‐reconditioning cycle was observed on mRNA expression of GPX3 (Figure 5b) and FABP4 expression (Figure 5c). Expression of GPX3, a ROS scavenger, was similar between CON and ASTX at DECON (*p* = 0.27). At RECON, GPX3 was upregulated in CON compared with DECON (*p* = 0.04) and was greater than ASTX (*p* = 0.05). Horses supplemented with ASTX had greater FABP4 than CON after DECON (*p* = 0.05). After RECON, expression of FABP4 was upregulated in CON relative to following DECON (*p* = 0.04). There was no observed treatment effect on FABP4 at RECON (*p* = 0.10).

## 4 DISCUSSION

To our knowledge, this study represents the first attempt to investigate the effects of an ASTX‐containing supplement during a deconditioning‐reconditioning cycle in horses. Oral administration of ASTX for a duration of 32 weeks modified circulating antioxidant activity, regardless of DECON and RECON. Upregulation of expression of genes involved in mitochondrial respiration and biogenesis as well as redox signaling was elicited by ASTX supplementation. A deconditioning‐reconditioning cycle impacted muscle oxidative status and mitochondrial respiration, which may be at least in part driven by up‐ and down‐regulation of genes in associated pathways.

Previous studies investigating the effects of exogenous antioxidant supplementation with vitamins and trace minerals in horses have reported enhanced oxidative status in circulation and skeletal muscle by increasing antioxidant capacity and decreasing exercise‐induced oxidative stress (Latham et al., 2021; Nemec et al., 2021; White et al., 2016). Comparably, previous research with healthy active young men (23.0 ± 2.0 years) demonstrated that daily supplementation of 6 mg/d ASTX for 4 weeks increased basal glutathione (GSH) activity in whole blood compared with unsupplemented individuals (McAllister et al., 2022). Additionally, plasma total antioxidant status in response to exercise was elevated in an ASTX supplemented group of young soccer players (Djordjevic et al., 2012). In a similar manner, the effects of ASTX, as an indirect antioxidant, were observed in the current work where the horses supplemented with ASTX had greater plasma GPX activity before and after SETs regardless of DECON and RECON. However, plasma SOD activity following SETs was lesser in ASTX than CON. Catalyzing superoxide to hydrogen peroxide, SOD helps maintain the redox state (Juarez et al., 2008). Our findings suggest that ASTX, as a direct antioxidant, might scavenge a greater amount of superoxide, requiring less SOD activity to reduce superoxide to H_2_O_2_. The differing effects on the different antioxidants suggest that, in this case, ASTX may act upstream of GPX, mitigating the need for as much SOD activity.

We observed a reduction in protein carbonyl concentration in circulation before and after SETs in horses receiving ASTX compared with CON following RECON. This finding is in agreement with previous studies conducted in diabetic men (Ciaraldi et al., 2023) and hypercholesterolemic rabbits (White et al., 2016) that reported a decrease in circulating protein carbonyl concentration in response to ASTX supplementation. Astaxanthin, therefore, may reduce oxidative modifications in proteins, regardless of conditioning status (Djordjevic et al., 2012; Nemec et al., 2021). In the current work, MDA and 8‐OHdG concentrations were not impacted by ASTX, suggesting that at this level of supplementation there were no measurable impacts on lipid or DNA oxidation. Increases in MDA and 8‐OHdG are often observed as exercise duration is increased (Leaf et al., 1997; Radák et al., 2000). Supplementation of ASTX may provide protection as exercise duration is increased, which requires additional study.

Interestingly, when Thoroughbred horses were supplemented with 75 mg ASTX and 3000 mg L‐carnitine together for 8 weeks, serum CK activity was decreased 4 h after acute strenuous exercise compared with an unsupplemented group (Sato et al., 2015). In the current work, there was no observed effect of ASTX on serum CK activity at any time point, which might be explained by a difference in the sample size, the timing of blood sample collection, or the specific exercise protocol. Evaluating the chronic response in addition to the acute response of CK activity to a strenuous exercise bout may be important to better understand the effects of ASTX on exercise‐induced muscle damage and its recovery.

Oxidative status within skeletal muscle was affected by DECON and RECON, but not ASTX supplementation. In response to DECON, muscle SOD activity was greater after SET than at rest. This was not observed following RECON, suggesting that greater muscle SOD activity might be required to handle exercise‐induced oxidative stress following DECON. Abundantly located within the cytosol and extracellular space, GPX3 reduces H_2_O_2_ to form water or alcohol (Powers et al., 2022). Within Pre‐Ex, muscle GPX was decreased after DECON compared with Baseline and RECON, indicating impaired basal antioxidant capacity in skeletal muscle. Correspondingly, GPX activity was greater at Post‐Ex than Pre‐Ex following DECON, while SET did not affect GPX following RECON. However, GPX3 gene expression following SETs was greater in CON than ASTX following RECON. We suspect that RECON may allow skeletal muscle to utilize GPX more efficiently to scavenge ROS produced during SET, leading to improved post‐exercise muscle recovery.

Altogether, ASTX may enhance some antioxidant mechanisms in circulation, but not skeletal muscle, and therefore reduce ROS production and oxidative damage to molecules, especially proteins. Production of small amounts of ROS activates several cellular redox signaling pathways to initiate biological processes. The ROS regulation of these pathways involves H_2_O_2_‐mediated oxidation of cysteine residues within proteins (Rhee, 1979), which causes allosteric changes within the protein and thereby alters its function (Winterbourn & Hampton, 2008). While these ROS‐mediated oxidative modifications can be reversed by thioredoxin and glutaredoxin, an overproduction of H_2_O_2_ further oxidizes the proteins, leading to protein carbonylation (Schieber & Chandel, 2014), an irreversible post‐translational modification (Fedorova et al., 2014). Hence, it is critical to maintain H_2_O_2_ homeostasis. This could be accomplished by scavenging superoxide radicals, which can be catalyzed to H_2_O_2_ by SOD, or reducing H_2_O_2_ to water by other antioxidants such as GPX (Ighodaro & Akinloye, 2018). The observed decrease in SOD, increase in GPX, and decrease in protein carbonylation in circulation of ASTX supplemented individuals indicate that ASTX might mitigate superoxide radicals and H_2_O_2_ directly and indirectly, respectively, which may help reduce protein oxidation.

Recognized as a mitochondrial‐targeted antioxidant, ASTX binds to mitochondrial membranes (Hecht et al., 2021). Previous reports have demonstrated that ASTX increased the expression of mitochondrial OXPHOS‐related genes (Krestinina et al., 2020; Mularczyk et al., 2022). However, there have been few studies investigating the effects of ASTX supplementation on skeletal muscle mitochondrial respiration. In the current work, only a deconditioning‐reconditioning cycle altered basal mitochondrial respiration capacity. State 3 respiration (oxidative phosphorylation), but not state 4 (leak) respiration, was altered, indicating that state 3 respiration drove changes in mitochondrial respiration efficiency. Importantly, athletes may experience more substantial skeletal muscle mitochondrial adaptations through physical activity rather than ASTX supplementation.

To gain further insights into the effects of ASTX on oxidative status and mitochondrial respiration during a deconditioning‐reconditioning cycle, we evaluated gene expression involved in redox balance and mitochondrial OXPHOS in muscle biopsy samples collected at Pre‐ and Post‐Ex. Our finding of upregulated basal expression of PPARGC1A induced by ASTX following RECON is consistent with the previous studies using a mouse model (Nishida et al., 2020). A knockout of PPARGC1A in mouse skeletal muscle results in downregulated gene expression of SHDB and MDH2, regulators of complex 2 function (Hatazawa et al., 2016), indicating the necessity of PPARGC1A in the induction of SHDB and MDH2. Thus, greater expression of MDH1, MDH2, SDHB, and SDHC in ASTX that was observed in the present study might result from greater PPARGC1A expression. The upregulated mRNA expression of PPARGC1A, however, did not induce expression of downstream genes involved in mitochondrial fusion and fission, MFN1 and DNM1, respectively. Contrarily, mice supplemented with ASTX during 6 weeks of high‐intensity interval training upregulated MFN1 and MFN2 expression (Wang et al., 2023). Notably, 10 mg/kg BW ASTX was given in olive oil to the mice while the horses in our study were supplemented with approximately 0.15 mg/kg BW ASTX mixed in a concentrate feed, which suggests that a greater amount of ASTX might be required to promote mitochondrial biogenesis‐related gene expression (Furuhashi et al., 2014; Numao et al., 2023).

In addition to the antioxidant properties, ASTX has been reported to promote basal lipid metabolism or FA oxidation (Aoi et al., 2008). We report that ASTX had greater FABP4 expression than CON in response to SET or following DECON. Greater basal FABP4 concentration in circulation is associated with the pathogenesis of metabolic disorders (Furuhashi et al., 2014). However, an increase in FABP4 content after strenuous SET has been reported both in trained and untrained men (Numao et al., 2023). Previous work by Iso et al. has reported that FABP4 deficient mice decreased running distance (Iso et al., 2019). Taken together, our finding of greater FABP4 in ASTX following DECON may suggest that horses supplemented with ASTX may have improved exercise endurance capacity via greater energy supply in skeletal muscle especially after a prolonged period of physical inactivity; however, this requires additional study.

While this study has provided insights into the effects of ASTX, deconditioning, and reconditioning, certain limitations should be acknowledged. For instance, previous studies have detected ASTX in the skeletal muscle of rodents following oral administration (Aoi et al., 2003; Choi et al., 2011). However, due to physiological differences between rodents and horses, it will be important to assess the bioavailability of ASTX across various species. In addition, the limited number of males in our sample precluded a comprehensive exploration of potential interactions between ASTX supplementation and sex. Whether supplementation of ASTX impacts male and female athletes differently has not yet been investigated. No rider information was collected during this study. Rider characteristics may impact RECON and performance in SET. However, the same riders were used for RECON and the SETs. Seasonal differences could impact the responses to the SET, as the DECON SET was performed in July, with higher ambient temperatures than in April and October (Baseline and RECON SETs, respectively).

### 4.1 Perspectives and significance

Our findings demonstrate that ASTX supplementation enhances circulating GPX capacity regardless of conditioning status, but did not affect other measures of muscle or circulating oxidative status. We also demonstrated that DECON and RECON impact skeletal muscle oxidative status and mitochondrial respiration, likely through alterations of related gene expression. Altogether, our results suggest that conditioning status has greater impacts on oxidative capacity than ASTX supplementation.

## AUTHOR CONTRIBUTIONS

M. Y. K. and S. A. R. conceived and designed research; M. Y. K., O. S. K., A. A., A. S. R., N. M. T., S. G. N., and S. A. R. performed experiments; M. Y. K., J. W. L., and T. E. M. analyzed data; M. Y. K. and S. A. R. interpreted results of experiments; M. Y. K. prepared figures; M. Y. K. drafted manuscript; M. Y. K., O. S. K., A. A., A. S. R., N. M. T., S. G. N., J. W. L., T. E. M, and S. A. R. edited and revised manuscript; M. Y. K., O. S. K., A. A., A. S. R., N. M. T., S. G. N., J. W. L., T. E. M, and S. A. R. approved final version of manuscript.

## FUNDING INFORMATION

This study was funded by a University of Connecticut Research Excellence Program grant to S. A. Reed.

## CONFLICT OF INTEREST STATEMENT

No conflicts of interest, financial or otherwise, are declared by the authors.

## ETHICS STATEMENT

All animal handling and experimental procedures followed the ethical guidelines and were approved by the Institutional Animal Care and Use Committee of the University of Connecticut.

## Supporting information


Tables S1–S3.

## Data Availability

The data that support the findings of this study are available from the corresponding author upon reasonable request.
