# Supplementary material for: Effects of an astaxanthin‐containing supplement on oxidative status in skeletal muscle and circulation during deconditioning and reconditioning periods in polo ponies
Source: Physiol Rep. 2025 Apr 26;13(8):e70346. doi: 10.14814/phy2.70346 (PMC12032451; doi:10.14814/phy2.70346)
Supplement: Supplementary file 1 — Tables S1–S3. [file PHY2-13-e70346-s001.docx]

SUPPLEMENTAL MATERIAL

**Supplementary Table 1.** Reconditioning program.

| Week | Walk (min) | Trot (min) | Canter (min) |
| --- | --- | --- | --- |
| 1 – 3 | 30 | 10 | - |
| 4 – 5 | 20 | 20 | 3 |
| 6 – 7 | 20 | 30 | 10 |
| 8 – 16 | 15 | 20 | 15 |

**Supplementary Table 2.** Nutrient composition^1^.

| Nutrient^2^ | ProElite Senior Concentrate | Timothy Grass Mix Hay | Mineral Block |
| --- | --- | --- | --- |
| DE, Mcal/kg | 3.08 | 2.01 | -- |
| CP, % | 17.8 | 10.8 | -- |
| ADF, % | 20.3 | 41.3 | -- |
| NDF, % | 30.4 | 64.1 | -- |
| Ca, % | 1.31 | 0.36 | 12.5 |
| P, % | 0.65 | 0.22 | 8.0 |
| Mg, % | 0.38 | 0.19 | 1.0 |
| K, % | 1.32 | 2.05 | 0.00003 |
| Na, % | 0.51 | 0.03 | 8.6 |
| Fe, ppm | 519 | 235 | -- |
| Zn, ppm | 305 | 19.0 | 2,200 |
| Se, ppm | -- | -- | 16 |
| Cu, ppm | 57.0 | 6.00 | 500 |
| ^1^Nutrient composition of concentrate and hay was analyzed by Dairy One, Inc. (Ithaca, NY). Mineral block information is based on the guaranteed analysis.  ^2^All nutrients are presented on a 100% dry matter basis. | | | |

Supplementary Table 3. Primer sequences.

| Gene^1^ |  | Primer sequences (5' → 3') | Amplicon length | Reference |
| --- | --- | --- | --- | --- |
| ATP5F1D |  |  |  |  |
| Forward |  | TACCTCCAAGTACTTTGTGAGCAGC | 201 | XM_023646356.1 |
| Reverse |  | TTGGCCTCGATGCGGATTTG |  |  |
| COX4I1 |  |  |  |  |
| Forward |  | GGGAGAAGCACTATGTGTACGG | 170 | XM_023637444.1 |
| Reverse |  | ATATGGAACGGCAGGTGCTT |  |  |
| COX4I2 |  |  |  |  |
| Forward |  | GATGAATCGCCGCTCCAATG | 122 | NM_001270956.1 |
| Reverse |  | GGGTGATGGGCTTCTTAGGG |  |  |
| CPT1A |  |  |  |  |
| Forward |  | AAGGGCGCTACTTCAAGGTC | 162 | NM_001081808.1 |
| Reverse |  | TGGCCCAGGGTACTCTTTCT |  |  |
| DNM1 |  |  |  |  |
| Forward |  | AACCAGCAACTGACGAACCA | 163 | XM_023629011.1 |
| Reverse |  | ACTGCTGGACCATCTGAAGC |  |  |
| FABP3 |  |  |  |  |
| Forward |  | TGGAAAACTCATCCTGACACTC | 141 | NM_001163885.2 |
| Reverse |  | ATGAGGCAATCTGGTGCTGAG |  |  |
| FABP4 |  |  |  |  |
| Forward |  | TACATGAAAGAAGTGGGAGTGGG | 151 | XM_005613035.3 |
| Reverse |  | CCTGGCCCAATTTGAAGGAAA |  |  |
| GPX3 |  |  |  |  |
| Forward |  | ACAGGAGAAGTCGAAGACGGA | 156 | NM_001115158.1 |
| Reverse |  | GTCAAGCCTCAGTAGCTGGC |  |  |
| MDH1 |  |  |  |  |
| Forward |  | CGCAGTTCCCAATCATGTCTGA | 180 | XM_001494265.5 |
| Reverse |  | CCATTAGGACACCGTCCAGG |  |  |
| MDH2 |  |  |  |  |
| Forward |  | GCACCTCAGCCCAGAACAAT | 233 | NM_001195526.2 |
| Reverse |  | CACATCGCAACCTTTCAGGC |  |  |
| MFN1 |  |  |  |  |
| Forward |  | CCTTGCCCCGAGTAGCATAA | 237 | XM_023623336.1 |
| Reverse |  | CGAGACAGCACCTCACCAAT |  |  |
| MFN2 |  |  |  |  |
| Forward |  | CAAGCTGAGACGGGACAGAG | 117 | XM_023635775.1 |
| Reverse |  | AGCACGGACATTGCGTTTTT |  |  |
| NDUFA9 |  |  |  |  |
| Forward |  | GGTCAATCACCTCGGACGTA | 201 | XM_001494601.4 |
| Reverse |  | GTTTCCCATTCTCGCCCAAC |  |  |
| NFE2L2 |  |  |  |  |
| Forward |  | TGTCGCGGAGAAGCGAGT | 185 | XM_023622057.1 |
| Reverse |  | ATGTCAATCAAATCCATGTCCTGC |  |  |
| OPA1 |  |  |  |  |
| Forward |  | GGCCCTGGACTACAGAGGAT | 178 | XM_005601892.3 |
| Reverse |  | TGCTACGTTCAGCATCCACA |  |  |
| PPARGC1A |  |  |  |  |
| Forward |  | AAGCTTCCCTCGAGATGCAAT | 232 | XM_014738763.2 |
| Reverse |  | AGAGCCAATGGCACATGGAA |  |  |
| PPARγ |  |  |  |  |
| Forward |  | GGGTGTCAGTTTCGCTCAGT | 156 | XM_023619952.1 |
| Reverse |  | ATTCATCAAGGAGGCCAGCA |  |  |
| SDHA |  |  |  |  |
| Forward |  | CAGTTCCACCCTACAGGCAT | 105 | XM_023625909.1 |
| Reverse |  | CTCCATGAACCTTTCGCCCT |  |  |
| SDHB |  |  |  |  |
| Forward |  | GGATTGACACCAACCTCAGCA | 209 | NM_001163823.2 |
| Reverse |  | CTCGTAGAGCCCGTCCAGT |  |  |
| SDHC |  |  |  |  |
| Forward |  | CTGCGCTGTTGTTGAGACAT | 187 | XM_001503765.6 |
| Reverse |  | GGAAGAGACCAACTGTAGATAGTGA |  |  |
| SDHD |  |  |  |  |
| Forward |  | TTGCCTCTGCTCTGTCATGC | 195 | XM_023644802.1 |
| Reverse |  | AGACAATCCCTCAACTCGCT |  |  |
| SLC2A4 |  |  |  |  |
| Forward |  | CTTCACCTTGGTCTCGGTGTT | 231 | NM_001081866.2 |
| Reverse |  | CTGAAGAGCTCAGCCACGAT |  |  |
| SOD1 |  |  |  |  |
| Forward |  | AGGACGAAGAGAGGCATGTTG | 124 | NM_001081826.3 |
| Reverse |  | GTGCGGCCAATGATGGAATG |  |  |
| SOD2 |  |  |  |  |
| Forward |  | CTCAGATTGCTCTGCAGCCT | 121 | NM_001082517.2 |
| Reverse |  | GCGTCCAGCAATTTCCCTTT |  |  |
| TUBA1 |  |  |  |  |
| Forward |  | AGGTGCTGCTTTTACAGGGA | 157 | XM_001504174.5 |
| Reverse |  | AATGGACAGCTTGGGTCTGT |  |  |
| TxnRd2 |  |  |  |  |
| Forward |  | TCGGCTGAGCACATTGTCAT | 137 | XM_023648096.1 |
| Reverse |  | CCGACCACCAACGTTTTTCC |  |  |
| UCP2 |  |  |  |  |
| Forward |  | CCAACCTCATGACAGATGACCT | 230 | XM_023645751.1 |
| Reverse |  | CCAGGAACCCAAACGGAGAA |  |  |
| UCP3 |  |  |  |  |
| Forward |  | CGGCCTTCTATAAGGGCAAGG | 146 | XM_014741874.2 |
| Reverse |  | AGGTGGAGTGGACTAAGGGT |  |  |
| UQCRB |  |  |  |  |
| Forward |  | TCGGTCTCGGGTCAAAATGG | 123 | XM_001490414.5 |
| Reverse |  | TCATCTCGCATTAACCCCAGT |  |  |

^1^Abbreviations: ATP5F1D = ATP synthease periopheral stalk subunit D; COX4I1 = Cytochrome c oxidase subunit 4 isoform 1; COX4I2 = Cytochrome c oxidase subunit 4 isoform 2; CPT1A = Carnitine palmitoyltransferase 1A; DNM1 = Dynamin related protein 1; FABP3 = Fatty acid binding protein 3; FABP4 = Fatty acid binding protein 4; GPX3 = Glutathione peroxidase 3; MDH1 = Malate dehydrogenase 1; MDH2 = Malate dehydrogenase 2; MFN1 = Mitofusin 1; MFN2 = Mitofusin 2; NDUFA9 = NAHD ubiquinone oxidoreductase subunit A9; NFE2L2 = Nuclear factor erythroid2-related factor 2; OPA1 = Mitochondrial dynamin like GTPase; PPARGC1A = Peroxisome proliferator-activated receptor γ coactivator-1; PPARγ = Peroxisome-proliferator activated receptor; SDHA = Succinate dehydrogenase complex subunit A; SDHB = Succinate dehydrogenase complex subunit B; SDHC = Succinate dehydrogenase complex subunit C; SDHD = Succinate dehydrogenase complex subunit D; SLC2A4 = Solute carrier family 2 member 4; SOD1 = Superoxide dismutase 1; SOD2 = Superoxide dismutase 2; TUBA1 = Tubulin alpha-1A chain; TxnRd2 = Thioredoxin reductase-2; UCP2 = Uncoupling protein 2; UCP3 = Uncoupling protein 3; UQCRB = Ubiquinol cytochrome c reductase binding protein
